# Gender and age-specific reference intervals of common biochemical analytes in Chinese population: Derivation using real laboratory data

**DOI:** 10.2478/jomb-2019-0046

**Published:** 2020-09-02

**Authors:** Danchen Wang, Chaochao Ma, Yutong Zou, Songlin Yu, Honglei Li, Xinqi Cheng, Ling Qiu, Tengda Xu

**Affiliations:** 1 Peking Union Medical College & Chinese Academy of Medical Science, Peking Union Medical College Hospital, Department of Clinical Laboratory, Beijing, China; 2 Chinese Academy of Medical Sciences, Peking Union Medical College Hospital, Department of Health Care, Dongcheng District, Beijing, China

**Keywords:** reference interval, large data set, Chinese population, indirect sampling method, referentni interval, veliki skup podataka, kineska populacija, metoda indirektnog uzorkovanja

## Abstract

**Background:**

Indirect sampling methods are not only inexpensive but also efficient for establishing reference intervals (RIs) using clinical data. This study was conducted to select fully normal records to establish ageand gender-specific RIs for common biochemical analytes by laboratory data mining.

**Methods:**

In total, 280,206 records from 2014 to 2018 were obtained from Peking Union Medical College Hospital. Common biochemical analytes total protein, albumin, total bilirubin (TBil), direct bilirubin (DBil), alanine aminotransferase (ALT), glutamyltranspeptidase (GGT), alkaline phosphatase (ALP), aspartate aminotransferase (AST), lactate dehydrogenase (LDH), potassium, sodium, chlorine, calcium, urea, glucose, uric acid (UA), inorganic phosphorus, creatinine (Cr), total cholesterol, triglyceride, high-density lipoprotein cholesterol, and low-density lipoprotein cholesterol] were measured using an automatic analyzer. Sources of variation were identified by multiple regression analysis. The 2.5th and 97.5th percentiles were calculated as the lower and upper limits of the RIs, respectively

**Results:**

Gender was the major source of variation among the 13 common biochemical analytes with an rp > 0.15. In contrast to the value listed in the WS/T 404, nearly all RIs established in this study were significantly narrower. Furthermore, age-specific RIs should be determined for DBil, LDH, and urea, whereas gender-specific RIs are suggested for GGT, LDH, and urea.

**Conclusions:**

We recommend that gender-specific RIs should be established for ALT, AST, GGT, DBil, TBil, UA, and Cr as well as genderand age-specific RIs for urea and ALP. Through indirect sampling, ageand gender-specific RIs for common biochemical analytes were established and analyzed.

## Abstract (sr)

**Uvod:**

Metode indirektnog uzorkovanja nisu samo jeftine, već su i efikasne za uspostavljanje referentnih intervala (RI) korišćenjem velike količine kliničkih podataka. Ova studija je sprovedena sa ciljem da se odaberu potpuno normalni pojedinačni zapisi da bi se utvrdio RI za starost i pol za zajedničke biohemijske analite pomoću laboratorijski dobijenih podataka.

**Metode:**

Ukupno je uzeto 280.206 pojedinačnih rezultata u periodu od 2014. do 2018. godine iz bolnice Peking Union Medical College-a. Uobičajeni biohemijski analiti [ukupni protein, albumin, ukupni bilirubin (TBil), direktni bilirubin (DBil), alanin aminotransferaza (ALT), glutamiltranspeptidaza (GGT), alkalna fosfataza (ALP), aspartat aminotransferaza (AST), laktat dehidrogenaza (LAT) kalijum, natrijum, hlor, kalcijum, ureja, glukoza, mokraćna kiselina (UA), neorganski fosfor, kreatinin (Cr), ukupni hole sterol, trigliceridi, lipoprotein holesterola visoke gustine i lipoprotein holesterola niske gustine] mereni su korišćenjem automatskog analizatora. Izvori varijacije identifikovani su višestrukom regresionom analizom. Postoci (2,5 i 97,5) su izračunati kao donja i gornja granica RI, svaki ponaosob.

**Rezultati:**

Pol je bio glavni izvor varijacije među 13 uobičajenih biohemijskih analita sa rp > 0,15. Za razliku od vrednosti navedene u WS / T 404, skoro svi RI utvrđeni u ovoj studiji bili su znatno uži. Dalje, za DBil, LDH i ureju trebalo bi odrediti RI specifične za starost, dok su za GGT, LDH i ureu, starosni referentni intervali predloženi.

**Zaključak:**

Preporučujemo da se utvrde referentni intervali specifični za pol za ALT, AST, GGT, DBil, TBil, UA i Cr, kao i referentni intervali specifični za pol i starost za ureju i ALP. Kroz indirektno uzorkovanje utvrđeni su i analizirani referentni intervali specifični za starosnu dob i za zajedničke biohemijske analite.

## Introduction

As parameters routinely used for clinical interpretation, reference intervals were first described in 1969 [1] and defined as prediction intervals that include the central 95% of reference values [2]. It is necessary to establish RIs for common analytes at large three-level general hospitals because the concentration of a given analyte may be affected by various factors such as gender, age, and ethnicity, among others [3]. Numerous studies have reported common RIs based on traceable results that can be used for analytes with good traceability from different manufacturers to the same higher-order references [4] [5] [6]. Reliable RIs are determined in completely healthy individuals but show variations [7] [8] [9] [10]. It is critical but difficult for each clinical laboratory to establish RIs [11], as it is not easy to recruit healthy individuals and ensure pre-analysis, mid-analysis, and post-analysis quality control. According to Clinical & Laboratory Standards Institute (CLSI) C28-A3 guidelines, two methods should be used to establish RIs: direct and indirect sampling methods [3] [12]. Establishing RIs by recruiting healthy individuals is a direct sampling method, which is costly and time-consuming [10] [13] [14]. First described in 1963 [15], indirect sampling must include a large number of normal individual records from databases, which is based on data mining techniques, cost-effective, and easier to perform in less time with lower material resources [7] [16] compared to direct sampling methods.

Accordingly, the aim of this study was to enroll completely normal records, establish age-and gender-specific RIs for common biochemical analytes, and compare the RIs with those listed in WS/T 404 [17] [18] [19] [20] [21] [22] [23].

## Materials and Methods

### Source data

In total, 280,206 records from 2014 to 2018 were obtained from the Peking Union Medical College Hospital. Each record included height, weight, systolic blood pressure (SBP), diastolic blood pressure (DBP), heart rate, nutrition status, past disease history, current symptoms, heart rhythm, abdominal palpation results, and basic biochemical measurements results. Basic laboratory measurements were collected including total protein (TP), albumin (Alb), total bilirubin (TBil), direct bilirubin (DBil), alanine aminotransferase (ALT), glutamyltranspeptidase (GGT), alkaline phosphatase (ALT), aspartate aminotransferase (AST), lactate dehydrogenase (LDH), potassium (K), sodium (Na), chlorine (Cl), calcium (Ca), urea, glucose (Glu), uric acid (UA), inorganic phosphorus (IP), creatinine (Cr), total cholesterol (TC), triglyceride (TG), high-density lipoprotein cholesterol (HDL-C), and low-density lipoprotein cholesterol (LDL-C). All common biochemical analytes were measured on a Roche C8000 automatic biochemical analyzer (Basel, Switzerland). Bodyweight was measured on a calibrated beam scale, and body mass index (BMI) was calculated as body weight divided by the square of the height (kg/m^2^). Blood pressure was measured after the participants had rested quietly for at least 20 min. All testing records associated with internal quality control, external quality assessment, and other interfering factors during this period were reviewed. All results were anonymized before analysis.

### Definition of the reference population

To establish RIs, we first defined »apparently healthy« individuals according to the protocol provided by the IFCC Committee on Reference Intervals and Decision Limits [24]. The following participants were excluded: (1) patients with acute or chronic diseases that required medical intervention including respiratory diseases, circulatory system disorders, liver or kidney diseases, acute and chronic infections, metabolic and nutritional disorders, autoimmune diseases, endocrine diseases, hematological diseases, and malignant tumor; 2) patients with BMI ≥28 or ≤18.5 kg/m^2^; (3) patients with an SBP of ≥160 mmHg or DBP of ≥100 mmHg; and (4) patients with incomplete biochemical tests.

A total of 148,332 (52.9%) participants were enrolled in this study. This study was approved by the Ethics Committee of Peking Union Medical College Hospital of the Chinese Academy of Medical Sciences. As this study was retrospective in nature and the results were anonymized, informed consent for the use of samples was not required.

### Definition of »fully normal individuals« by secondary exclusion

Secondary exclusion of candidate reference individuals was performed according to the Tukey method to exclude outliers. A previous study revealed that Alb, UA, Glu, HDL-C, LDL-C, TG, AST, ALT, and GGT represented changes in nutrition status and metabolic syndrome (the most prevalent latent disease) [25]. Thus, we used the Tukey method to exclude abnormal tests results. A total of 125,509 (44.8%) individuals considered as fully normal individuals were enrolled in the final analysis.

### Statistical analysis

Data were analyzed using SPSS 20.0 software (SPSS, Inc., Chicago, IL, USA). Multiple regression analysis (MRA) was performed to identify sources of variation (SVs) potentially affecting common biochemical analytes. A standardized partial regression coefficient (r_p_) of >0.15 was considered practically significant, corresponding to P < 0.0001 with a large sample size of approximately 1500. Analysis of variance or the Mann-Whitney U test was performed to evaluate the significance of differences between subgroups, such as gender and age [26] [27]. Using a nonparametric method, RIs were calculated as the 2.5^th^ percentile confidence interval (P_*2.5*_) and 97.5^th^ percentile confidence interval (P_*97.5*_) according to CLSI C28-A3 [12].

## Results

### Basic characteristics of enrolled fully normal individual records

A total of 125,509 fully normal individual records (male: n = 55.885; female: n = 69.624) were enrolled in this study (Table 1).

**Table 1:** Characteristics of fully normal individual records ^a^: P values between male and female

	Male	Female	P-value	Total
N (%)	55885 (44.5%)	69624 (55.9%)	----	125509
Age, years	38.8 ± 10.9	38.9 ± 10.6	0.385	38.8 ± 10.7
BMI, kg/m^2^	23.9 ± 2.2	22.2 ± 2.3	< 0.001	22.9 ± 2.4
SBP, mmHg	120 ± 14	110 ± 14	< 0.001	115 ± 15
DBP, mmHg	75 ± 9	68 ± 9	< 0.001	71 ± 9

### SVs of RIs of common biochemical analytes

MRA was performed to evaluate the SVs of common biochemical analytes. The results are shown in Table 2. Gender was the major SV for Alb, TBil, DBil, ALT, GGT, ALP, AST, Na, Ca, urea, UA, Cr, and HDL-C (r_p_> 0.15). Age was also an important SV for TP, AST, LDH, Na, Cl, Ca, urea, Glu, TC, TG, and LDL-C. BMI was associated with ALT, GGT, TG, HDL-C, and LDL-C. However, SBP and DBP showed no significant association with any common biochemical analytes.

**Table 2:** Source of variations in RIs of common biochemical analytes (rp) R represents the multiple correlation coefficient. Values shown are standardized partial regression coefficients (rp). Values of (rp) ≥ 0.15 that were significant are marked in bold letters

Analyte	R	Gender	Age	BMI	SBP	DBP
TP, g/L	0.269	0.007	-0.222	-0.058	0.099	0.110
Alb, g/L	0.366	-0.385	-0.051	-0.044	-0.017	-0.018
TBil, µmol/L	0.309	-0.312	-0.037	-0.090	0.028	0.011
DBil, µmol/L	0.3	-0.300	-0.059	-0.128	0.048	-0.013
ALT, U/L	0.472	-0.313	0.023	0.216	0.019	0.042
GGT, U/L	0.498	-0.330	0.102	0.193	-0.020	0.095
ALP, U/L	0.338	-0.185	0.143	0.090	0.107	-0.009
AST, U/L	0.327	-0.216	0.180	0.043	0.056	-0.002
LDH, U/L	0.314	-0.059	0.173	0.099	0.148	-0.010
K, mmol/L	0.063	0.017	0.021	0.057	0.019	-0.028
Na, mmol/L	0.349	-0.276	0.182	0.008	0.061	-0.029
Cl, mmol/L	0.233	0.110	0.161	0.077	-0.069	-0.058
Ca, mmol/L	0.283	-0.172	-0.159	-0.050	0.109	0.039
Urea, mol/L	0.36	-0.308	0.192	0.018	0.020	-0.048
Glu, mmol/L	0.366	-0.060	0.216	0.120	0.116	0.040
UA, µmol/L	0.668	-0.557	-0.085	0.181	0.033	0.020
IP, mmol/L	0.31	0.220	-0.126	-0.075	-0.001	-0.036
Cr, µmol/L	0.771	-0.767	0.019	0.012	0.010	-0.011
TC, mmol/L	0.311	0.050	0.267	0.066	0.020	0.066
TG, mmol/L	0.451	-0.134	0.172	0.257	-0.018	0.128
HDL-C, mmol/L	0.485	0.294	0.045	-0.295	0.033	-0.038
LDL-C, mmol/L	0.363	-0.059	0.249	0.151	0.007	0.069

### Distribution of common biochemical analytes by gender and age

The distribution of common biochemical analytes of fully healthy individuals according to gender are shown in Table 3. The median TBil, DBil, ALT, GGT, ALP, AST, LDH, urea, UA, Cr, TG, HDL-C, and LDL-C values in males were significantly higher than those in females. TP, Alb, K, Na, Cl, IP, Ca, Glu, and TC were significantly different between males and females. Common biochemical analyte levels by age are shown in Table 4. The serum levels of GGT, LDH, urea, Glu, Cr, TC, TG, and LDL-C increased with age, whereas HDL-C levels decreased with age. The ALP levels were sharply increased in patients older than 50 years of age.

**Table 3:** Distribution of common biochemical analytes by gender LL: lower limit; UL: upper limit

Analyte	Total			Male			Female		
	Median	LL	UL	Median	LL	UL	Median	LL	UL
TP, g/L	73	66	80	73	66	80	73	66	80
TBil, µmol/L	11	5	26	13	6	28	10	4	22
DBil, µmol/L	4.1	2.1	8.3	4.5	2.4	8.9	3.7	2.0	7.5
ALT, U/L	15	7	38	19	9	42	13	6	31
GGT, U/L	16	8	45	21	10	49	13	7	37
ALP, U/L	58	35	98	63	40	99	54	33	97
AST, U/L	17	11	28	19	12	29	16	11	27
LDH, U/L	162	121	219	166	126	220	158	119	217
K, mmol/L	4.2	3.7	4.9	4.2	3.7	4.8	4.2	3.7	4.9
Na, mmol/L	141	137	144	141	138	144	140	137	144
Cl, mmol/L	102	98	106	102	97	106	102	98	106
IP, mmol/L	1.15	0.87	1.44	1.11	0.84	1.40	1.19	0.92	1.46
Ca, mmol/L	2.37	2.20	2.54	2.39	2.23	2.56	2.35	2.19	2.53
Urea, mol/L	4.34	2.60	6.90	4.71	3.05	7.22	4.03	2.45	6.48
Glu, mmol/L	5.0	4.3	6.0	5.1	4.3	6.1	5.0	4.3	5.9
UA, mol/L	295	177	475	355	238	506	255	166	375
Cr, mol/L	69	49	100	83	66	105	61	47	78
TC, mmol/L	4.5	3.2	6.4	4.6	3.2	6.3	4.5	3.2	6.4
TG, mmol/L	0.95	0.44	2.50	1.12	0.50	2.68	0.84	0.42	2.22
HDL-C, mmol/L	1.36	0.86	2.15	1.21	0.81	1.91	1.49	0.96	2.24
LDL-C, mmol/L	2.80	1.63	4.44	2.93	1.73	4.48	2.69	1.57	4.40

**Table 4:** Distribution of common biochemical analytes by age LL: lower limit; UL: upper limit.

Analyte	19–29 years			30–39 years			40–49 years			50–64 years			≥65 years		
	median	LL	UL	median	LL	UL	median	LL	UL	median	LL	UL	median	LL	UL
TP, g/L	74	67	81	73	66	80	72	65	79	72	65	79	72	64	80
TBil, µmol/L	11	5	27	11	5	26	10	5	25	10	5	24	11	5	25
DBil, µmol/L	4.3	2.2	8.8	4.1	2.1	8.3	3.9	2.0	7.9	3.9	2.1	7.8	4.2	2.3	8.7
ALT, U/L	14	6	39	15	7	39	16	7	38	17	9	37	15	8	33
GGT, U/L	14	7	39	15	8	44	16	8	47	18	9	47	17	9	43
ALP, U/L	57	34	95	57	34	96	57	35	94	66	40	106	67	40	109
AST, U/L	17	11	27	17	11	28	17	12	28	19	13	30	20	13	30
LDH, U/L	159	121	213	158	120	212	162	121	216	173	130	232	182	136	251
K, mmol/L	4.2	3.7	4.9	4.2	3.7	4.8	4.2	3.7	4.8	4.2	3.7	4.9	4.3	3.7	4.9
Na, mmol/L	140	137	144	140	137	144	140	137	144	141	138	145	142	138	145
Cl, mmol/L	102	97	106	102	98	106	102	98	106	103	98	107	103	98	107
IP, mmol/L	1.21	0.93	1.48	1.15	0.88	1.43	1.12	0.86	1.40	1.15	0.85	1.44	1.10	0.83	1.40
Ca, mmol/L	2.40	2.24	2.57	2.37	2.20	2.54	2.34	2.18	2.52	2.36	2.20	2.54	2.35	2.19	2.54
Urea, mol/L	4.18	2.50	6.67	4.23	2.56	6.64	4.36	2.62	6.93	4.71	2.92	7.33	5.11	3.18	8.21
Glu, mmol/L	4.9	4.2	5.7	5.0	4.3	5.8	5.1	4.3	6.0	5.2	4.4	6.3	5.3	4.5	6.3
UA, µmol/L	303	185	484	295	177	479	285	168	467	296	183	460	305	190	483
Cr, µmol/L	70	49	101	69	48	100	69	48	100	70	49	100	75	51	108
TC, mmol/L	4.25	3.07	5.91	4.42	3.16	6.12	4.65	3.30	6.36	5.00	3.46	6.84	4.95	3.25	6.86
TG, mmol/L	0.80	0.41	2.09	0.91	0.44	2.45	1.02	0.47	2.59	1.18	0.54	2.68	1.19	0.56	2.65
HDL-C, mmol/L	1.40	0.88	2.15	1.34	0.85	2.12	1.35	0.85	2.14	1.35	0.85	2.20	1.37	0.84	2.26
LDL-C, mmol/L	2.52	1.51	4.04	2.72	1.60	4.28	2.92	1.75	4.47	3.21	1.92	4.82	3.14	1.67	4.77

### RIs of common biochemical analytes

Based on their SVs and distribution, the RIs for common biochemical analytes were calculated. All RIs established in this study were significantly narrower than those given by the WS/T 404 [17] [18] [19] [20] [21] [22] [23] (Supplementary Table 1). The ranges of TP, ALT, GGT, ALP, AST, K, Na, Cl, and IP in this study were significantly lower, whereas the distributions of Alb, TBil, and Ca were higher than those suggested by the WS/T 404. We recommended establishing genderspecific RIs for ALT, AST, GGT, DBil, TBil, UA, and Cr and gender-and age-specific RIs for urea and ALP.

**Supplementary Table 1:** Comparation reference interval of common biochemical analytes between this study and WS/T 404

Analyte	Unit	Sex	Age	Reference interval
Urea	mmol/L	Male	20–59	3.1–8.0
	mmol/L	Male	60–79	3.9–9.5
	mmol/L	Female	20–59	2.6–7.5
	mmol/L	Female	60–79	3.1–8.8
Cr	µmol/L	Male	20–59	57–97
	µmol/L	Male	60–79	57–111
	µmol/L	Female	20–59	41–73
	µmol/L	Female	60–79	41–81
Ca	mmol/L	Total	None	2.11–2.52
IP	mmol/L	Total	None	0.85–1.51
Mg	mmol/L	Total	None	0.75–1.02
LD	U/L	Total	None	120–250
CK	U/L	Male	None	50–310
	U/L	Female	None	40–200
AMY	U/L	Total	None	35–135
TP	g/L	Total	None	65–85
Alb	g/L	Total	None	40–55
GLB	g/L	Total	None	20–40
K	mmol/L	Total	None	3.5–5.3
Na	mmol/L	Total	None	137–147
Cl	mmol/L	Total	None	99–110
TBil	µmol/L	Male	None	≤26
	µmol/L	Female	None	≤21
	µmol/L	Total	None	≤23
DBIL	µmol/L	Total	None	≤8
ALT	U/L	Male	None	9–50
	U/L	Female	None	7–40
ALT (with 5’ pyridoxal phosphate）	U/L	Male	None	9–60
	U/L	Female	None	7–45
AST	U/L	Male	None	15–40
	U/L	Female	None	13–35
AST (with 5 pyridoxal phos-	U/L	Male	None	15–45
	U/L	Female	None	13–40
ALP	U/L	Male	None	45–125
	U/L	Female	20–49	35–100
	U/L	Female	50–79	50–135
GGT	U/L	Male	None	10–60
	U/L	Female	None	7–45

## Discussion

Common biochemical analytes are very important for evaluating the health condition of individuals, and reliable RIs are needed for physicians to interpret test results and diagnoses. Many clinical laboratories directly use RIs from foreign manufacturers or those found in the scientific literature; however, many factors including different ethnicity, analyzers, gender, and age can affect the distribution of biochemical analytes [14]. Therefore, it is important to identify fully normal individual records and establish RIs for common biochemical analytes. Accordingly, an indirect sampling method and nonparametric analysis were applied to establish RIs for common biochemical analytes in this study.

As suggested by the results of MRA, gender was the major SV for 13 common biochemical analytes, whereas age was an important SV for 11 analytes. We found that gender-specific RIs should be established for ALT and AST, which have narrow RIs according to reported guidelines [17] [18] [19] [20] [21] [22]. ALP levels were sharply increased in patients greater than 50 years old, which is consistent with the results of a previous study [24] [17] [18] [19] [20] [21] [22]. In this study, we found that serum GGT levels in males were higher than those in females. A nationwide Chinese population-based cross-sectional study revealed that men had a higher serum level of GGT than women in each quartile [27]. The RIs of GGT in this study were 10-49 U/L (male) and 7-37 U/L (female), which are narrower than the range for WS/T (10-60 U/L (male), 7-45 U/L (female)) [17] [18] [19] [20] [21] [22]. A nationwide multicenter study in China reported that the RIs of GGT were 11-65 U/L (male) and 8-36 U/L (female) [24]. Thus, we recommend establishing gender-specific RIs for GGT (r_p _= -0.330). This study revealed that age-or gender-specific RIs are unnecessary for K, Na, Cl, Ca, and IP, as no clinically significant differences were observed, which is consistent with the results of previous studies [24] [17] [18] [19] [20] [21] [22]. Based on the results described above, the RIs of common biochemical analytes were established in this study, most of which were significantly narrower than those listed in the WS/T 404 [17] [18] [19] [20] [21] [22]. Additionally, the ranges of TP, ALT, GGT, ALP, AST, K, Na, Cl, and IP in this study were significantly lower, while the distributions of TBil, and Ca were higher than those in the WS/T 404.

There were several advantages in this study. Firstly, few studies have established RIs for common biochemical analytes using the indirect sampling method [13] [28]. Use of the direct sampling method requires the recruitment of healthy individuals ratherthan directly collecting big clinical data, which is timeconsuming and costly [29]. In contrast, using an indirect sampling method is much easier and more costefficient. We also included a large cohort of patients for whom health checkups were performed for five years at the same facility to reduce inter-assay variation. Otherwise, all measurements were performed according to our laboratory standard operating procedures. Our laboratory also takes part in external quality assessments by the National Center for Clinical Laboratories and the College of American Pathologists to guarantee the accuracy and reliability of our study [9].

In this study, we identified the main SVs contributing to the distribution of common biochemical analytes using the MRA method and established ageor gender-specific RIs using an indirect sampling method. We recommend that gender-specific RIs should be established for ALT, AST, GGT, DBil, TBil, UA, and Cr, as well as gender-and age-specific RIs for urea and ALP. Using an indirect sampling method may enable more laboratories to establish their own RIs, particularly for specific populations such as the elderly and children. Guidelines for determining standard RIs based on big clinical data are needed.

*Acknowledgements*. This work was funded byresearch grants from the National Natural ScienceFoundation of China (grant number 81702060) (http://www.nsfc.gov.cn/).

## Conflict of interest statement

The authors state that they have no conflicts of interest regarding the publication of this article.

## List of abbreviations

TP, total protein; Alb, albumin; TBil, totalbilirubin; DBil, direct bilirubin; ALT, alanine aminotransferase; GGT, glutamyl transferase; ALP, alkaline phosphatase; AST,aspartate aminotransferase; LDH, lactate dehydrogenase; K,potassium; Na, sodium; Cl, chlorine; Ca, calcium; Glu, glucose; UA, uric acid; IP, inorganic phosphorus; Cr, creatinine; TC, total cholesterol; TG, triglyceride; HDL-C, high-densitylipoprotein cholesterol; LDL-C, low-density lipoprotein cholesterol; SVs, source of variation. 
